# ge-CRISPR - An integrated pipeline for the prediction and analysis of sgRNAs genome editing efficiency for CRISPR/Cas system

**DOI:** 10.1038/srep30870

**Published:** 2016-09-01

**Authors:** Karambir Kaur, Amit Kumar Gupta, Akanksha Rajput, Manoj Kumar

**Affiliations:** 1 Bioinformatics Centre, Institute of Microbial Technology, Council of Scientific and Industrial Research, Sector 39A, Chandigarh-160036, India

## Abstract

Genome editing by sgRNA a component of CRISPR/Cas system emerged as a preferred technology for genome editing in recent years. However, activity and stability of sgRNA in genome targeting is greatly influenced by its sequence features. In this endeavor, a few prediction tools have been developed to design effective sgRNAs but these methods have their own limitations. Therefore, we have developed **“ge-CRISPR”** using high throughput data for the prediction and analysis of sgRNAs genome editing efficiency. Predictive models were employed using SVM for developing pipeline-1 (classification) and pipeline-2 (regression) using 2090 and 4139 experimentally verified sgRNAs respectively from *Homo sapiens, Mus musculus, Danio rerio* and *Xenopus tropicalis*. During 10-fold cross validation we have achieved accuracy and Matthew’s correlation coefficient of 87.70% and 0.75 for pipeline-1 on training dataset (**T**^**1840**^) while it performed equally well on independent dataset (**V**^**250**^). In pipeline-2 we attained Pearson correlation coefficient of 0.68 and 0.69 using best models on training (**T**^**3169**^) and independent dataset (**V**^**520**^) correspondingly. **ge-CRISPR** (http://bioinfo.imtech.res.in/manojk/gecrispr/) for a given genomic region will identify potent sgRNAs, their qualitative as well as quantitative efficiencies along with potential off-targets. It will be useful to scientific community engaged in CRISPR research and therapeutics development.

Clustered regularly interspaced short palindromic repeats (CRISPR) and CRISPR associated (Cas) proteins are machineries that deliver adaptive immunity to prokaryotes (bacteria and Achaea)^1^,^2^. They work together to identify and destroy foreign nucleic acids^3^,^4^. CRISPRs are short repeats, which are parted with spacer sequences of viral or foreign origin^5^. These repeat spacer elements are reported in almost all sequenced archaeal and half of the bacterial genomes^6^. Immediately adjacent to the CRISPR loci there are present different cas genes that are involved in acquisition and invading of foreign nucleic acid^7^. On the basis of signature cas genes CRISPR system has been grouped into 3 classes i.e. Type I, Type II and Type III^8^. Type II CRISPR/Cas system from *Streptococcus pyogenes* is now used widely for CRISPR/Cas mediated genome engineering, as it requires only a single protein Cas9^8^. Cas9 enzyme contains two nuclease domains - HNH domain that cleaves target i.e. complementary strand and RuvC domain splits non-complementary strand^10^. Based on the properties of Cas9, researchers have used it along with Crispr RNA (crRNA) and trans activating RNA (tracrRNA) complex to cleave segment of DNA and applied it for genome editing^11^.

Doudna *et al.* in 2012, published a seminal article demonstrating that it is feasible to engineer crRNA and tracrRNA to form chimeric single guide RNA (sgRNA) with better functionality and ease for genome editing^9^. sgRNA contains a 20 nucleotide complementary sequence to the target and can be designed according to any genomic sequence., After its binding with target, Cas9 is recruited to mediate double stranded cleavage at the target site adjacent to Protospacer adjacent motif (PAM)^9^. Subsequently, edited sites are repaired by cells internal DNA repair mechanisms like non-homologous end joining (NHEJ) and homology directed repair (HDR). NHEJ can lead to introduction of insertions/deletions (indels) of variable length at the target site that may change the reading frame of a coding sequence^12^. HDR can be utilized to create point mutations or to introduce desired segment at the target locus^13^.

Due to the efficiency and simplicity of sgRNA and Cas9, CRISPR/Cas9 has been successfully utilized to edit genomes of different life forms like humans, mice, nematodes, flies, plants etc.^14^,^15^,^16^,^17^. George Church *et al.* has demonstrated editing in human genome using CRISPR/Cas9 for the very first time^18^ for targeting AAVSI locus fragments. Subsequently, this technique was employed to engineer mouse embryos, and also to grow stem cells in culture^19^. Multiple sgRNAs has also been employed to prompt large inversions or deletions between the double stranded breaks in order to generate simultaneous mutations at more than three genes in Zebrafish^20^. Other studies have also highlighted the use of this approach in food crops like rice (*Oryza sativa*) and wheat (*Triticum aestivum*) targeting phytoene desaturase gene efficiently^21^.

This new technology also helped in culminating various genetic disorders and has a potential to create scar less modifications that was not possible earlier^22^. Apart from this, CRISPR has been effectively applied to alter genomes of viruses like Human immunodeficiency virus (HIV)^23^, Human papillomavirus (HPV)^24^, Epstein bar virus (EBV)^25^ etc. In addition to target DNA sequences CRISPR/Cas9 from *Francisella novicida* has been reprogrammed to alter viral RNA i.e +ssRNA virus (hepatitis C virus) in eukaryotic cells leading to inhibition of viral protein production^26^.

CRISPR/Cas based genome editing studies have been flourished in no time and hence lead to development of a few bioinformatics repositories. We have developed “CrisprGE” a central hub to provide comprehensive information of genome editing by this technique. This database harbors genes/genomes edited, organisms, experimental procedures and modifications along with its efficiency^27^. There is another depository “CRISPRz” just having details of genome editing in zebrafish^28^.

Computational approaches have helped the researchers in CRISPR/Cas based genome editing applications. First step in genome editing is the identification of sgRNA^29^, for which few tools have been developed namely GT-Scan^30^, CRISPRdirect^31^, SSFinder^32^ and E-CRISPR^33^ etc. These tools extract 20 nucleotides sequences adjacent to “NGG” PAM motif in the genomes as sgRNAs. In aditition to identification of sgRNAs more significant approaches are needed to access the activities of Cas9 at off-target sites i.e. sites having few nucleotides difference as compared to the target site. For this *in-silico* tools CasOT^34^ and Cas-OFFinder^35^ are available.

Although, above tools identify many sgRNAs for a particular gene/genome but not all sgRNAs have equal efficiency. Infact, their genome editing efficiency may range from least to very high. Therefore, algorithms for selecting highly specific and efficient sgRNA from a pool of identified sgRNAs are highly desired. Few methods have been developed to address this issue like sgRNAdesigner^36^, WU-CRISPR^37^, sgRNAScorer^38^ and CRISPRscan^39^. Amongst them, sgRNAdesigner^36^, WU-CRISPR^37^, sgRNAScorer^38^ classify library of sgRNAs on the basis of its high and low activity while CRISPRscan is dedicated to predict the actual efficacy of sgRNAs.

However, existing methods have certain shortcomings like these algorithms utilized in house datasets generated on a specific experimental setting. Present tools have exploited limited sequence features and their hybrids, also the set of sgRNA used in each study are different. Prediction tools of sgRNA used different methods for estimating efficacy i.e. some has employed readout assay and others have used phenotype based readout method to predict specificity and efficiency of a particular sgRNA. Existing methods are also limited to design sgRNAs against limited eukaryotic genomes like *Homo sapiens, Mus musculus, Danio rerio* and *Xenopus tropicalis* etc.

In the present study, we tried to address the inadequacies in the existing tools. For this we have compiled high throughput experimental data and explored numerous sgRNA sequence features, to see their effect on the activity of sgRNA. Further, we have developed Support vector machine (SVM) based predictive models to calculate qualitative and quantitative efficiency of sgRNAs better than the existing tools. The same is assimilated in a web server “**ge-CRISPR”** which is an integrated pipeline to predict sgRNAs with high on target and minimum off target activity. This algorithm will be very beneficial to select single sgRNA with high potency from a pool of identified sgRNAs.

## Results

By complying data from diverse platforms we have generated 2 pipelines i.e. pipeline-1, which classify sgRNAs into high and low potency and pipeline-2 quantifies calculates actual efficiency of a particular sgRNA. Employing several sgRNA sequence features and using SVM, we have generated predictive models and results of which are specified below.

### Performance evaluation of pipeline-1 (geCRISPRc) predictive models during 10-fold cross validation and on Independent datasets

In ge-CRISPR various sgRNA sequence features have been employed to develop predictive models namely composition profile, binary profile, secondary structure, thermodynamic features and their hybrids.

In composition profile i.e. mono, di, tri, tetra and penta nucleotides we achieved accuracy and Mathew’s correlation coefficient (MCC) of 65.60%, 66.30%, 73.26%, 72.45%, 65.82% and 0.32, 0.35, 0.47, 0.45, 0.33 respectively. We have also checked performance of various hybrid composition features like mono-di, mono-di-tri, mono-di-tri-tetra, and mono-di-tri-tetra-penta nucleotides. Hybrid features provided accuracy and MCC of 79.57%, 82.17%, 81.85%, 80.65% and 0.60, 0.64, 0.64, 0.61 correspondingly. Hybrid composition features performed better than their individual ones.

Comparably, for binary profile we achieved enhanced performance in individual features *viz.* mono and di (1-2-3 degree) rather than their hybrids. Accuracy and MCC in case of mono, di (1-2-3 degree) binary are 85.54%, 87.17%, 84.57%, 83.70% and 0.71, 0.75, 0.70, 0.68 respectively. Similarly, thermodynamics and secondary structure features attained accuracy and MCC of 75.38%, 0.51, and 53.07%, 0.10 correspondingly. Among various features employed, di-binary performed best and secondary structure least. Further we also analyzed different hybrid features, detailed results of all features are shown in Table 1.

Besides 10-fold cross validation, independent dataset was used to evaluate the final performance of the models as described in Table 1. Amongst all, the best performing feature in training/testing i.e. di (1-degree) binary profile performed equally well on independent dataset with accuracy and MCC of 88.80% and 0.78 correspondingly.

### ROC plot for validating threshold independent performance of hybrid models

**Figure 1:**
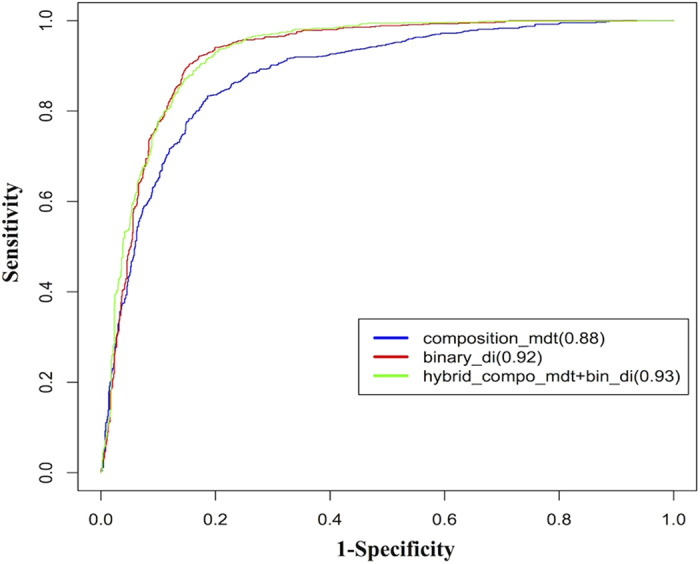
ROC representing Area under the curve between different hybrids features. In composition profile hybrid of mono-di-trinucleotide have AUC of 0.88 (blue), binary profile of dinucleotide have AUC of 0.92 (red) and hybrid of mono-di-trinucleotide composition and dinucleotide binary display AUC of 0.93 (green).

To check the threshold independent performance of various hybrid models, receiver operating characteristic (ROC) curve was plotted using R. ROC displays area under the curve (AUC) which is formed by plotting sensitivity (true positive rate or recall) against 1-specificity (false positive rate or precision). It determines which of the used models predicts the given classes best. AUC values of above mentioned features ranges from 0.54–0.93 and the best values were obtained for hybrids of composition mono-di-tri (0.88); dinucleotide (1-degree) binary (0.92) and hybrid of composition mono-di-tri and dinucleotide (1-degree) binary (0.93). AUC of 1 for a model determines its goodness therefore; models with higher AUCs are preferred more than with lower AUCs. ROC plot of hybrids is depicted in Fig. 1.

### Performance evaluation of pipeline-2 (geCRISPRr) predictive models during 10-fold cross validation and on independent datasets

We have utilized 22 sequence features to calculate the quantitative efficiency of sgRNA using SVM. During 10-fold cross validation on training/testing (**T**^**3619**^) dataset, we obtained PCC of 0.32, 0.33, 0.44, 0.44 and 0.34 respectively for mono, di, tri, tetra and penta nucleotides. Whereas hybrids features like mono-di, mono-di-tri, mono-di-tri-tetra, and mono-di-tri-tetra-penta nucleotides increased PCC to 0.52, 0.58, 0.58 and 0.57 respectively. Likewise in classification, predictive models based on regression performed best on binary profile and increased PCC to 0.68 at di (1 degree) and also with mono-di (1 degree) binary feature. Thermodynamic properties exhibited correlation of 0.44 and secondary structure did not perform well. Apart from these we also checked the performance of all hybrid features, which are briefed in Table 2.

In addition to 10-fold cross validation, we used 520 sgRNAs for independent validation of the models. All the predictive models performed equally well on independent datasets. Best models of composition and binary profiles displayed PCC of 0.56 and 0.69 respectively. It shows that predictive models are well trained and can be employed to predict efficiency of unknown sgRNA. Results of all the sgRNA features on validation dataset are summarized in Table 2.

### Analysis of sgRNAs using nucleotide position

**Figure 2:**
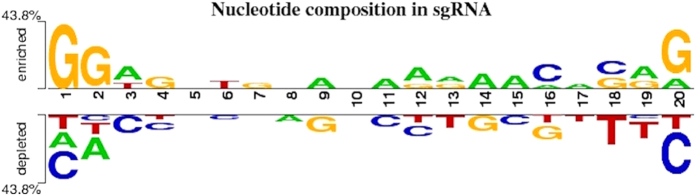
Two sample sequence logo depicting preference of A,T,G,C at 20 positions of highly effective and least effective sgRNAs.

In order to analyze which nucleotides are favoured in sgRNAs, we studied the frequency of each of the four nucleotides i.e A, T, C, and G at 20 positions of sgRNA upstream to PAM using two sample logos. We have utilized 1021 positive (having >50% efficiency) and 1069 negative (<5% efficiency) sgRNA sequences. Positional analysis revealed that Guanine is favoured at 1^st^, 2^nd^ and 20^th^ position followed by Cytosine at 16^th^ and 18^th^ position and Adenine at maximum positions. Thymidine has minimum propensity of occurrence and found only at 5^th^ and 3rd position shown in Fig. 2. In consistent with previous studies^36^,^39^, we also observed depletion of cytosine and enrichment of guanine at 20^th^ position of sgRNA.

### Web server

geCRISPR is freely accessible at http://bioinfo.imtech.res.in/manojk/gecrispr/. In order to predict the best sgRNA, user need to input desired gene or genome sequence in fasta format. The output displays potential sgRNA with PAM, start and end position, GC percentage and potency. We have integrated 2 pipelines that calculate efficiency both qualitatively as well as quantitatively along with off-targets associated with that particular sgRNA. Integrated pipeline for the prediction and analysis of sgRNA genome editing efficiency operates in 4 steps i.e sgRNA scanner, pipeline-1, pipeline-2 and off-target analysis.

#### sgRNA scanner

For this we have developed in-house perl script. User is required to paste single or multiple genes/genomic segments in FASTA format in the text box provided. sgRNA scanner identifies putative sgRNAs by scanning the user provided gene/genome in both sense and antisense strand on the basis of NGG PAM sequence. It then displays all possible sgRNAs (20-nucleotide) upstream to PAM. One can select from any of the given pipelines i.e. pipeline-1 and pilpeline-2.

##### ***Pipeline-1*** (geCRISPRc)

It will help to classify sgRNA scanned by sgRNA scanner into high and low potency (qualitatively). Output display serial no. of sgRNA, sgRNA sequences, PAM, start and end coordinates, GC%, sgRNA potency respectively. By clicking on an individual entry it will display complete sgRNA profile along with the secondary structure, sgRNA map and off-targets for that particular sgRNA as depicted in Supplementary Fig. S1.

##### ***Pipeline-2*** (geCRISPRr)

**Figure 3:**
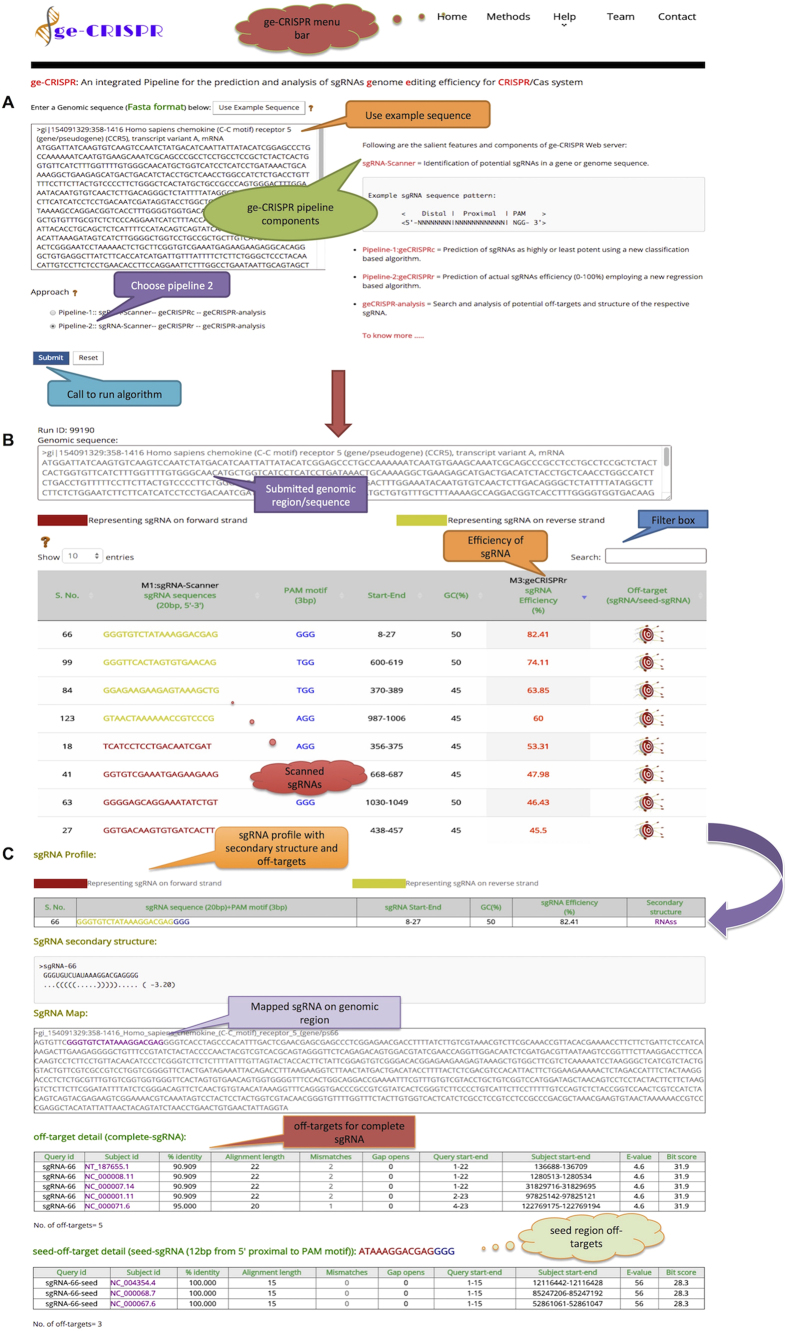
Workflow of pipeline-2. (**a**) sgRNA scanner for extracting sgRNAs in user provided genome/gene (**b**) output of pilpeline-2 depicting efficiency of each sgRNA (**c**) sgRNA profile, displaying secondary structure and off-targets associated with individual sgRNA.

This algorithm will help to quantitatively predict the actual efficiency of sgRNA in terms of percentage (0–100%). Similar to pipeline-1 output of pipeline-2 exhibit serial no. of sgRNA, sgRNA sequences, PAM, start and end coordinates, GC% but instead of qualitative potency it will calculate the quantitative efficiency of an sgRNA for genome editing (Fig. 3a,b).

#### Off-target analysis

Efficient sgRNA should target on desired site (on-target) with no or partial homologous sites (off-targets) elsewhere in the target genome. An analysis tool is integrated to screen on/off-targets for the particular sgRNA in the model organisms i.e*. Homo sapiens, Mus musculus, Drosophila melanogaster, and Denio rerio*. In this study, standalone blast (blastn algorithm) was used to map putative sgRNAs to whole genomes.

This is accomplished in two modes:

##### Complete-sgRNA searching mode

Using the complete sgRNA sequence of 23nt including NGG (PAM) motif to evaluate potential off-target sites in both strands of target genome with the search criteria i.e. percent identity with target should be more than 90% (~1 or 2 mismatch allowed).

##### Seed-sgRNA (~12nt + 3nt (PAM)) searching mode

In this, only a seed region^29^ (i.e. 12nt 5′ proximal to the NGG PAM motif) of particular sgRNA is utilized to screen potential off-target in both strands of target genome. As any mismatch in seed region is the most critical determinant of specificity than the distal (non-seed) ~8 nucleotides therefore, 100% sequence identity parameter was used.


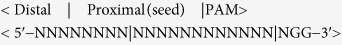


Off-target analysis is represented in two ways. First, as tabular output, that integrates information of Query-ID, Subject-ID, Percent Identity, Alignment length, Mismatches, Gap opens, Query start-end, subject start-end, e-value and score. Second, it provides detailed output with mapped sequence of query sgRNAs and potential off-targets with the details of gaps and mismatches as illustrated in Fig. 3c.

### Comparison with existing sgRNA prediction algorithms

As already mentioned, a few tools for predicting the efficiency of sgRNA have been developed. We have compared models generated by us with existing servers. These web servers are developed using their in house data and in comparison we have utilized available high throughput data on a single platform to build universal model which may be applicable to all. In addition to this we have performed both classification and regression analysis that is not done before. We compared our ge-CRISRc with the algorithm developed in classification mode e.g. sgRNA designer, WU-CRISPR and sgRNAScorer. Moreover, for regression analysis we compared geCRISPRr with CRISPRscan. We have employed various sequence features and results of our best models are shown in Tables 3, 4 while details of all features used are given in Supplementary Tables (S1–4). Our algorithms (classification and regression) performed better than existing ones despite having data of different platforms.

## Discussion

Genome editing remains an important scientific endeavor to make desired changes in the genomic regions^40^. It has been accomplished successfully using various nucleases like like zinc fingers nucleases (ZFs) and transcription activator-like effector nucleases (TALENs) etc^41^,^42^. Initial methods used customizable DNA binding domains fused with FokI nuclease domain that cuts non-specifically. Although these approaches have made several progresses but still have their own limitations i.e. need for engineering new enzyme for every target site, which is very costly and time consuming. In 2012 Doudna *et al.* have demonstrated the use of bacterial CRISPR-Cas9 system in genome editing for the first time^9^. This method provides specificity and multiplicity, which is far beyond the capacity of the earlier methods. Due to its ease this method has also been selected as the “Breakthrough of the year” in 2015 by Science magazine^43^.

To access the ability of CRISPR/Cas for genome editing identification of sgRNA adjacent to NGG PAM motif is the first step. For which several *Insilco* tools i.e. GT-Scan^30^, CRISPRdirect^31^, SSFinder^32^ and E-CRISPR^33^ have been developed. These tools although identify putative sgRNAs in a genome but does not predict their editing efficiency. Subsequently, focus shifted on to develop such algorithms, which can predict the actual efficiency of sgRNA.

A few tools have been developed to design active sgRNAs but on different platforms. For example, sgRNAdesigner^36^ is a method in which authors have constructed predictive models to calculate activity of sgRNAs against 6 human and 3 mice genes. By utilizing their dataset (1841) we developed SVM models and achieved better accuracy and MCC of 97.28% and 0.95 respectively (Table 3, Supplementary Table S1). Later on, Wong *et al.* developed another webserver WU-CRISPR^37^ using same dataset with AUC of 0.92. Our geCRISPRc method performed better with AUC of 0.99 even in comparison to WU-CRISPR. In another study, Chari *et al.*^38^ have also analyzed sequence features to identify sgRNA activity of ~1400 genes of human and mice with accuracy of 73.2% (Cas9_sp_) and 81.5% (Cas9_st1_). Again on the same dataset (Cas9_sp_ 279, Cas9_st1_171), we obtained comparatively better results with an accuracy of 76.7% and 83.44% correspondingly (Table 3, Supplementary Tables S2–3). Finally, we compiled diverse dataset of sgRNAs from different platforms in geCRISPRc and managed a better performance with an accuracy and MCC of 81.17% and 0.75 respectively (Table 3).

Simultaneously, Mateos *et al.* have developed CRISPRScan^39^ a regression based method for predicting actual efficiency of an sgRNA instead of just classifying it as either effective or non-effective. This tool was developed using in-house 1020 sgRNAs against 128 genes of *Danio rerio* and attained a correlation of 0.45 on training dataset. We have exploited this dataset also and developed a regressive model that attained similar performance. Contrary to this, our geCRISPRr algorithm based on larger dataset (3619) achieved far better performance with PCC of 0.68 (Table 4, Supplementary Table S4).

We have also cross checked the performance of earlier predictive models (developed on a particular dataset) on another experimental sgRNA dataset. We observed that predictors which are made on similar platforms performed well on each other but they did not achieve better results otherwise. Therefore, there is an irresistible need to develop algorithms using sgRNA datasets from diverse experimental conditions. Here, we attempted to develop generalized predictive SVM models that works on heterogeneous data of experimental sgRNAs in both classification and regression mode.

For ge-CRISPR, we have exploited various sgRNA sequence features like compositional profile, binary profile, thermodynamic properties, secondary structure and their hybrids. Amongst these binary features performed best followed by thermodynamic and compositional profile, whereas secondary structure did not achieved satisfactory results. Moreover, hybrid features achieved similar performance to that of binary. Of the binary features explored, 1-degree binary have shown highest accuracy and correlation. It could be attributed to the fact that in 1-degree binary both composition and neighbouring residue positions are taken into account simultaneously. Similar observations of binary features have also been reported by others.

Apart from the development of SVM based predictions these models have been integrated into a user-friendly web server i.e. “ge-CRISPR”. It works in a pipeline like it firstly scan genomes on the basis of “NGG” motif and extract 20 nucleotides upstream to this motif. This will limit off target upto some extent as unique 20 nucleotides target sequence and NGG will not occur more likely on other locations of the genome. After extraction of all possible sgRNAs another algorithm geCRISPRc will differentiate these sgRNAs into high and low potency along with its GC content and start-end position in the genome. geCRISPRr will allow users to have quantitative estimate of an individual sgRNA by calculating its efficiency in range of 0–100%. Additionally, off target tool is also incorporated to further know off targets associated with sgRNA in the seed region or in complete sgRNA if any. This integrated pipeline will surely help to have highly active sgRNA for intended targeting of genomes prior to experimental validation.

### Future developments and conclusion

We will further update our algorithm to increase its predictive potential once enough data accumulates. It would be interesting in future to explore the role of other features like dynamical modeling or spatial effects^44^,^45^,^46^ etc. to analyze activity of sgRNAs. For example Sciabola *et al.* have used 3D structural information of siRNA duplex as a descriptor to determine the activity of siRNA^47^. Lu *et al.* have also developed a tool to explore and annotate 3D structures of molecules like viral tRNA mimic, SAM-1 riboswitch, Cas9-sgRNA-DNA etc.^48^.

The performance of ge-CRISPR pipeline is better than earlier methods as it holds potential to predict both quantitative and qualitative efficiencies of sgRNAs for diverse organisms. We foresee that this integrated pipeline will help the scientific community in accelerating CRISPR based genome editing and therapeutics.

## Methods

### Data Curation

**Figure 4:**
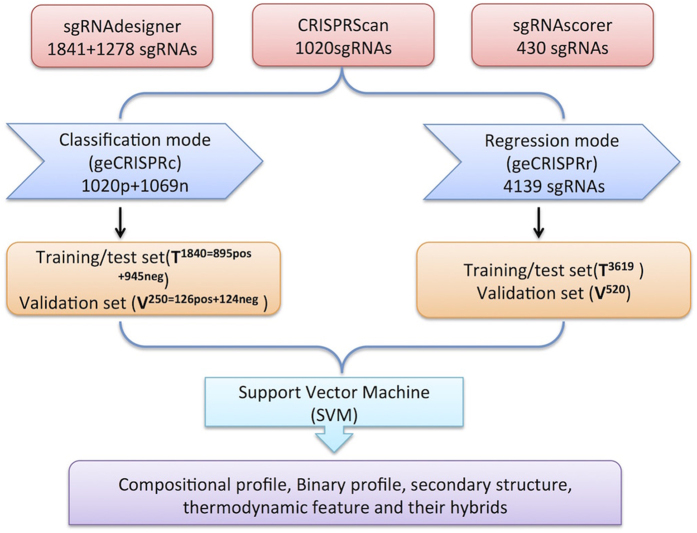
Diagrammatic representation of ge-CRISPR pipeline development.

We have extracted sgRNA sequences from different studies namely sgRNAdesigner (1841 and additional 1278)^36^, CRISPRScan (1020)^39^ and sgRNAscorer (430)^38^. For sgRNA designer, data was freely available in supplementary information in the form of sgRNA sequences (1841, 1278) and their respective scores. For sgRNAScorer, data was available in supplementary information as high and low activity sgRNAs (Cas9_sp_ 279, Cas9_st1_171). For CRISPRscan, the author has kindly provided the dataset with sgRNA sequences (1020) and efficiency on our request. Overall data was categorized for classification and regression studies. In classification mode, total of 2090 sgRNAs (including potent 1021 sgRNAs having >50% efficiency and 1069 sgRNAs with <5% efficiency) were used. Further, overall sgRNA sequences were divided into Training/Testing (**T**^**1840=895pos+945neg**^) and Validation (**V**^**250=126pos+124neg**^) datasets. In regression mode, 4139 sgRNAs (efficiency from 0 to 100%) were splitted into Training/Testing (**T**^**3619**^) and Validation (**V**^**520**^) datasets. Workflow of geCRISPR is represented in Fig. 4.

#### sgRNA sequence features

In this algorithm, we have employed different parameters of sgRNA sequence (20 nucleotides excluding PAM) features like composition profile, binary profile, secondary structure features, thermodynamic properties and their hybrids. These features were reported to have correlation with the activity of sgRNAs^39^.

#### Composition profile

Composition profile is the frequency of each nucleotide in an sgRNA i.e. A, T, G, C. The main purpose of calculating nucleotide composition is to change alterable length of sequences into a fixed length vector^49^. This is the critical step as any machine learning technique (MLT) involves vectors of fixed length. We explored mono to penta nucleotide compositional profiles corresponding to 4, 16, 64, 256, and 1024 vectors respectively.

#### Binary profile

Earlier studies have utilized positions of nucleotides to calculate the activity of sgRNAs^37^. We also studied binary profiles to extract features of sgRNAs on the basis of occupancy of nucleotides in sgRNA. Different binary patterns were used i.e. mono-binary and di-binary (1, 2, 3 degree). In mono-binary profile, probability of occurrence of each nucleotide (A, T, G or C) at every position was studied. Moreover, for di-binary profile, sequences were examined on basis of 1 degree in which probability of occurrence of all contiguous residues was calculated. Additionally, for 2 degree and 3 degree di-nucleotides with all 2^nd^ and 3^rd^adjacent residues were evaluated.

#### Thermodynamic properties

Thermodynamic stability of nucleotides is important as mentioned in previous studies on siRNA and sgRNA^37^,^50^. We have also incorporated this feature to check the thermodynamic stability of sgRNA sequences. In total 20 features were combined to calculate Gibbs free energies of different sets of interacting nucleotides.

#### Secondary structure property

Secondary structure of RNA represents the ability of a molecule to form intramolecular bonds for its stabilization. We calculated equilibrium base-pairing probabilities and minimum free energy (MFE) of sgRNA sequences^39^ by utilizing RNA fold from Vienna RNA package^51^. Output is represented in the form of bracket and dots that symbolize pairing or unpairing of nucleotides respectively. These features were further converted into binary values i.e. 1or 0 for SVM input.

#### Hybrid approaches

Despite using the above-mentioned features individually, in past hybrid approaches were explored^49^. In this algorithm we have also studied different hybrid features of composition and binary profile along with thermodynamics and secondary structure. Detailed list of all the features used is listed in Table 1.

#### Support Vector Machine

Support Vector machine (SVM) is a supervised MLT used for classification and regression analysis based on statistical and optimization theory. *SVM*^*light*^ (v6.02) (http://svmlight.joachims.org/) package was used for developing predictive models in various algorithms like QSPpred^52^, AVPpred etc.^53^. It develops models by classifying patterns in training/testing data that helps to consign categories to unidentified sequences. We used Radial basis function (RBF) kernel on varied g and c values.

#### 10-fold cross validation

For evaluating performance of all modules, we performed 10-fold cross validation. In this approach, the whole dataset is randomly divided into equally sized ten sets. Further, from this one set is used for testing and the remaining nine sets are considered for training and this process is then iterated ten times so that each dataset is being tested once.

#### Performance parameters

Performance of various models was calculated using threshold dependent and independent parameters. In threshold dependent module, we analysed performance in terms of 1) Sensitivity, 2) Specificity, 3) Accuracy and 4) MCC as calculated using following equations:

##### Sensitivity

This parameter measures the test’s capability to correctly predict positive sgRNAs from actual positive sgRNAs.


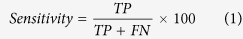


where, TP is correctly predicted positive sgRNAs and FN is falsely predicted negative sgRNAs.

##### Specificity

Specificity refers to test’s ability to correctly predict negative sgRNAs from actual negative sgRNAs.


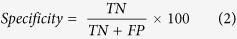


where, TN is correctly predicted negative sgRNAs and FP is falsely predicted positive sgRNAs.

##### Accuracy

Accuracy determines the success of correctly predicted sgRNAs from overall data. It defines the closeness of predicted results to the true value and the reproducibility of the same prediction.


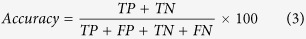


##### Mathew’s correlation coefficient (MCC)

MCC is a measure of calculating correlation between the observed and the predicted classification. It reconciles the imbalance between positive and negative datasets. Its value ranges from −1 to +1 where +1 signifies perfect correlation, 0 depicts no well than random correlation and -1 signifies total divergence between predicted and observed classification.


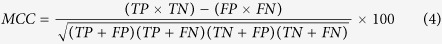


In threshold independent module we employed ROC to study performance of various SVM models. We generated ROC plots depicting area under curve (AUC) using ROCR statistical package available in R^54^.

Sometimes another parameter of evaluation is used called as Pearson’s correlation coefficient (R). This predicts the correlation between the actual and the predicted data i.e. how the change in one variable is affecting the other variable linearly. The value of correlation ranges from perfect correlation of +1 to total negative correlation of −1.


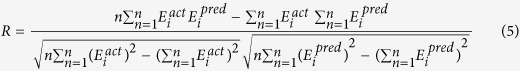


where n represents size of the test set while Ei^pred^ and Ei^act^ are the predicted and actual efficiencies of sgRNAs respectively.

### Two Sample Logo (TSL)

TSL was created using online Two Sample Logo tool^55^. This tool helps to graphically represent position specific differences in nucleotides between positive and negative dataset statistically. TSL contains a stack of symbols in which height of each stack represents relative frequency of a nucleotide whereas height of symbol indicates sequence conservation at that position^52^. In this study, we have utilized TSL to analyze the preference of nucleotides on the 20 positions of sgRNA. Upper section of this displays enrichment of nucleotides and lower sections signify depletion of a particular nucleotide in the given dataset at a particular position.

## Additional Information

**How to cite this article**: Kaur, K. *et al.* ge-CRISPR - An integrated pipeline for the prediction and analysis of sgRNAs genome editing efficiency for CRISPR/Cas system. *Sci. Rep.* **6**, 30870; doi: 10.1038/srep30870 (2016).

## Supplementary Material

Supplementary Information

## Figures and Tables

**Table 1:** Performance of pipeline-1 (geCRISPRc) predictive models on sgRNA sequences (T^1840^) using Support vector machine during 10-fold cross validation and on Independent datasets (V^250^).

S.No	sgRNA features	Vector	T^1840^				V^250^	
			Acc	MCC	AUC	Acc	MCC	AUC
1	Mononucleotide composition	4	65.6	0.32	0.70	69.2	0.39	0.73
2	Dinucleotide composition	16	66.3	0.35	0.73	69.2	0.40	0.76
3	Trinucleotide composition	64	73.26	0.47	0.81	75.2	0.51	0.82
4	Tetranucleotide composition	256	72.45	0.45	0.80	74	0.49	0.81
5	Pentanucleotide composition	1024	65.82	0.33	0.71	68.8	0.39	0.73
6	1 + 2	20	79.57	0.6	0.86	85.2	0.71	0.92
7	1 + 2+3	84	82.17	0.64	0.88	87.2	0.75	0.92
8	1 + 2 + 3 + 4	340	81.85	0.64	0.88	83.6	0.69	0.92
9	1 + 2 + 3 + 4 + 5	1364	80.65	0.61	0.87	82.8	0.66	0.9
10	Mononucleotide binary	80	85.54	0.71	0.92	90	0.8	0.95
**11**	**Dinucleotide (1-degree) binary**	**304**	**87.17**	**0.75**	**0.92**	**88.8**	**0.78**	**0.94**
12	Dinucleotide (2-degree) binary	288	84.57	0.70	0.90	86.4	0.73	0.93
13	Dinucleotide (3-degree) binary	272	83.7	0.68	0.90	86	0.72	0.93
14	10 + 11	384	77.23	0.58	0.78	90.4	0.81	0.95
15	11 + 12 + 13	864	86.47	0.73	0.92	89.6	0.79	0.95
16	10 + 11 + 12 + 13	944	86.96	0.74	0.93	88.8	0.78	0.95
17	Secondary structure	20	53.7	0.10	0.54	58	0.16	0.59
18	Thermodynamic features	21	75.38	0.51	0.82	74.4	0.49	0.82
19	7 + 16	1028	86.41	0.73	0.93	91.2	0.82	0.95
20	7 + 16 + 18	1049	83.7	0.68	0.91	84.4	0.70	0.93
21	7 + 16 + 17	1048	86.25	0.73	0.93	90.4	0.81	0.95
22	7 + 16 + 17 + 18	1069	83.48	0.67	0.91	83.6	0.67	0.92

Acc, accuracy; MCC, Matthew’s correlation coefficient; AUC, area under curve.

**Table 2:** Performance of pipeline-2 (geCRISPRr) predictive models on Training/testing (T^3619^) and independent datasets using (V^250^) Support vector machine during 10-fold cross validation.

S.No	sgRNA features	Vector	T^3619^	V^520^
			PCC	PCC
1	Mononucleotide composition	4	0.32	0.31
2	Dinucleotide composition	16	0.33	0.31
3	Trinucleotide composition	64	0.44	0.45
4	Tetranucleotide composition	256	0.44	0.44
5	Pentanucleotide composition	1024	0.34	0.36
6	1 + 2	20	0.52	0.48
7	1 + 2 + 3	84	0.58	0.54
8	1 + 2 + 3 + 4	340	0.58	0.56
9	1 + 2 + 3 + 4 + 5	1364	0.57	0.54
10	Mononucleotide binary	80	0.65	0.67
11	Dinucleotide (1degree) binary	**304**	**0.68**	**0.69**
12	Dinucleotide (2degree) binary	288	0.59	0.60
13	Dinucleotide (3degree) binary	272	0.60	0.61
14	10 + 11	384	0.68	0.69
15	11 + 12 + 13	864	0.65	0.65
16	10 + 11 + 12 + 13	944	0.65	0.65
17	Secondary structure	20	0.01	0.05
18	Thermodynamic features	21	0.44	0.50
19	7 + 14	468	0.66	0.63
20	7 + 14 + 17	488	0.12	0.60
21	7 + 14 + 18	489	0.66	0.63
22	7 + 14 + 17 + 18	509	0.12	0.60

PCC, Pearson correlation coefficient.

**Table 3:** Comparison of pipeline-1 (geCRISPRc) with existing sgRNA potency prediction algorithms.

Algorithm	Dataset Train/Test	Length of sgRNA	Acc	MCC	AUC	References
sgRNA designer	736 = 368p + 368n	20	NA	NA	NA	Doench *et al.* Nature Biotech. 2014
WU-CRISPR	736 = 368p + 368n	20	NA	NA	0.91	Wong *et al.* Genome Biology 2015
**geCRISPRc**	**736** = **368p** **+** **368n**	**20**	**97.28**	**0.95**	**1.0**	Our Study
sgRNAScorer_sp	279 = 133p + 146n	23	73.2	NA	NA	Chari *et al.* Nature Methods 2015
**geCRISPRc**	**279** = **133p** **+** **146n**	**23**	**76.7**	**0.54**	**0.82**	Our Study
sgRNAScorer_st	171 = 82p + 89n	27	81.5	NA	NA	Chari *et al.* Nature Methods 2015
**geCRISPRc**	**171** **=** **82p** **+** **89n**	**27**	**83.44**	**0.67**	**0.88**	Our Study
**geCRISPRc_overall**	**1840** **=** **895p** **+** **945n**	**20**	**87.17**	**0.75**	**0.92**	

ACC; accuracy, MCC; Matthew’s correlation coefficient, AUC; area under curve, NA; Not Available.

**Table 4:** Comparison of pipeline-2 (geCRISPRr) with existing sgRNA efficiency prediction algorithms.

Algorithm	Dataset Train/Test	PCC	Dataset Independent	PCC	Reference
CRISPRscan	1020	0.45	NA	0.58	Mateos *et al.* 2015, Nature Methods
**geCRISPRr**	1020	0.43	NA	NA	Our study
**geCRISPRr_overall**	**3619**	**0.68**	**520**	**0.69**	

PCC; Pearson correlation coefficient, NA; Not available.

## References

[b1] Ishino Y., Shinagawa H., Makino K., Amemura M. & Nakata A. Nucleotide sequence of the iap gene, responsible for alkaline phosphatase isozyme conversion in Escherichia coli, and identification of the gene product. J Bacteriol 169, 5429–5433 (1987). 3316184 10.1128/jb.169.12.5429-5433.1987 PMC213968

[b2] Marraffini L. A. & Sontheimer E. J. CRISPR interference: RNA-directed adaptive immunity in bacteria and archaea. Nat Rev Genet 11, 181–190, doi: 10.1038/nrg2749 (2010). 20125085 PMC2928866

[b3] Brouns S. J. *et al.* Small CRISPR RNAs guide antiviral defense in prokaryotes. Science 321, 960–964, doi: 10.1126/science.1159689 (2008). 18703739 PMC5898235

[b4] Gasiunas G., Barrangou R., Horvath P. & Siksnys V. Cas9-crRNA ribonucleoprotein complex mediates specific DNA cleavage for adaptive immunity in bacteria. Proc Natl Acad Sci USA 109, E2579–E2586, doi: 10.1073/pnas.1208507109 (2012). 22949671 PMC3465414

[b5] Pourcel C., Salvignol G. & Vergnaud G. CRISPR elements in Yersinia pestis acquire new repeats by preferential uptake of bacteriophage DNA, and provide additional tools for evolutionary studies. Microbiology 151, 653–663, doi: 10.1099/mic.0.27437-0 (2005). 15758212

[b6] Grissa I., Vergnaud G. & Pourcel C. The CRISPRdb database and tools to display CRISPRs and to generate dictionaries of spacers and repeats. BMC Bioinformatics 8, 172, doi: 10.1186/1471-2105-8-172 (2007). 17521438 PMC1892036

[b7] Haft D. H., Selengut J., Mongodin E. F. & Nelson K. E. A guild of 45 CRISPR-associated (Cas) protein families and multiple CRISPR/Cas subtypes exist in prokaryotic genomes. PLoS Comput Biol 1, e60, doi: 10.1371/journal.pcbi.0010060 (2005). 16292354 PMC1282333

[b8] Chylinski K., Makarova K. S., Charpentier E. & Koonin E. V. Classification and evolution of type II CRISPR-Cas systems. Nucleic Acids Res 42, 6091–6105, doi: 10.1093/nar/gku241 (2014). 24728998 PMC4041416

[b9] Jinek M. *et al.* A programmable dual-RNA-guided DNA endonuclease in adaptive bacterial immunity. Science 337, 816–821, doi: 10.1126/science.1225829 (2012). 22745249 PMC6286148

[b10] Mali P., Esvelt K. M. & Church G. M. Cas9 as a versatile tool for engineering biology. Nat Methods 10, 957–963, doi: 10.1038/nmeth.2649 (2013). 24076990 PMC4051438

[b11] Kirchner M. & Schneider S. CRISPR-Cas: From the Bacterial Adaptive Immune System to a Versatile Tool for Genome Engineering. Angew Chem Int Ed Engl 54, 13508–13514, doi: 10.1002/anie.201504741 (2015). 26382836

[b12] Ran F. A. *et al.* Genome engineering using the CRISPR-Cas9 system. Nat Protoc 8, 2281–2308, doi: 10.1038/nprot.2013.143 (2013). 24157548 PMC3969860

[b13] Li K., Wang G., Andersen T., Zhou P. & Pu W. T. Optimization of genome engineering approaches with the CRISPR/Cas9 system. PLoS One 9, e105779, doi: 10.1371/journal.pone.0105779 (2014). 25166277 PMC4148324

[b14] Upadhyay S. K., Kumar J., Alok A. & Tuli R. RNA-guided genome editing for target gene mutations in wheat. G3 (Bethesda) 3, 2233–2238, doi: 10.1534/g3.113.008847 (2013). 24122057 PMC3852385

[b15] Mashiko D. *et al.* Generation of mutant mice by pronuclear injection of circular plasmid expressing Cas9 and single guided RNA. Sci Rep. 3, 3355, doi: 10.1038/srep03355 (2013). 24284873 PMC3842082

[b16] Chen C., Fenk L. A. & de Bono M. Efficient genome editing in Caenorhabditis elegans by CRISPR-targeted homologous recombination. Nucleic Acids Res. 41, e193, doi: 10.1093/nar/gkt805 (2013). 24013562 PMC3814388

[b17] Ren X. *et al.* Optimized gene editing technology for Drosophila melanogaster using germ line-specific Cas9. Proc Natl Acad Sci USA 110, 19012–19017, doi: 10.1073/pnas.1318481110 (2013). 24191015 PMC3839733

[b18] Mali P. *et al.* RNA-guided human genome engineering via Cas9. Science 339, 823–826, doi: 10.1126/science.1232033 (2013). 23287722 PMC3712628

[b19] Yang H., Wang H. & Jaenisch R. Generating genetically modified mice using CRISPR/Cas-mediated genome engineering. Nat Protoc 9, 1956–1968, doi: 10.1038/nprot.2014.134 (2014). 25058643

[b20] Jao L. E., Wente S. R. & Chen W. Efficient multiplex biallelic zebrafish genome editing using a CRISPR nuclease system. Proc Natl Acad Sci USA 110, 13904–13909, doi: 10.1073/pnas.1308335110 (2013). 23918387 PMC3752207

[b21] Shan Q. *et al.* Targeted genome modification of crop plants using a CRISPR-Cas system. Nat Biotechnol 31, 686–688, doi: 10.1038/nbt.2650 (2013). 23929338

[b22] Xie F. *et al.* Seamless gene correction of beta-thalassemia mutations in patient-specific iPSCs using CRISPR/Cas9 and piggyBac. Genome Res. 24, 1526–1533, doi: 10.1101/gr.173427.114 (2014). 25096406 PMC4158758

[b23] Ebina H., Misawa N., Kanemura Y. & Koyanagi Y. Harnessing the CRISPR/Cas9 system to disrupt latent HIV-1 provirus. Sci Rep. 3, 2510, doi: 10.1038/srep02510 (2013). 23974631 PMC3752613

[b24] Hu Z. *et al.* Disruption of HPV16-E7 by CRISPR/Cas system induces apoptosis and growth inhibition in HPV16 positive human cervical cancer cells. Biomed Res Int 2014, 612823, doi: 10.1155/2014/612823 (2014). 25136604 PMC4127252

[b25] Yuen K. S. *et al.* CRISPR/Cas9-mediated genome editing of Epstein-Barr virus in human cells. J Gen Virol 96, 626–636, doi: 10.1099/jgv.0.000012 (2015). 25502645

[b26] Price A. A., Sampson T. R., Ratner H. K., Grakoui A. & Weiss D. S. Cas9-mediated targeting of viral RNA in eukaryotic cells. Proc Natl Acad Sci USA 112, 6164–6169, doi: 10.1073/pnas.1422340112 (2015). 25918406 PMC4434742

[b27] Kaur K., Tandon H., Gupta A. K. & Kumar M. CrisprGE: a central hub of CRISPR/Cas-based genome editing. Database (Oxford) 2015, bav055, doi: 10.1093/database/bav055 (2015). 26120138 PMC4483309

[b28] Varshney G. K. *et al.* CRISPRz: a database of zebrafish validated sgRNAs. Nucleic Acids Res. 44, D822–D826, doi: 10.1093/nar/gkv998 (2016). 26438539 PMC4702947

[b29] Hsu P. D. *et al.* DNA targeting specificity of RNA-guided Cas9 nucleases. Nat Biotechnol 31, 827–832, doi: 10.1038/nbt.2647 (2013). 23873081 PMC3969858

[b30] O’Brien A. & Bailey T. L. GT-Scan: identifying unique genomic targets. Bioinformatics 30, 2673–2675, doi: 10.1093/bioinformatics/btu354 (2014). 24860161 PMC4155256

[b31] Naito Y., Hino K., Bono H. & Ui-Tei K. CRISPRdirect: software for designing CRISPR/Cas guide RNA with reduced off-target sites. Bioinformatics 31, 1120–1123, doi: 10.1093/bioinformatics/btu743 (2015). 25414360 PMC4382898

[b32] Upadhyay S. K. & Sharma S. SSFinder: high throughput CRISPR-Cas target sites prediction tool. Biomed Res Int 2014, 742482, doi: 10.1155/2014/742482 (2014). 25089276 PMC4095993

[b33] Heigwer F., Kerr G. & Boutros M. E-CRISP: fast CRISPR target site identification. Nat Methods 11, 122–123, doi: 10.1038/nmeth.2812 (2014). 24481216

[b34] Xiao A. *et al.* CasOT: a genome-wide Cas9/gRNA off-target searching tool. Bioinformatics, doi: 10.1093/bioinformatics/btt764 (2014). 24389662

[b35] Bae S., Park J. & Kim J. S. Cas-OFFinder: a fast and versatile algorithm that searches for potential off-target sites of Cas9 RNA-guided endonucleases. Bioinformatics 30, 1473–1475, doi: 10.1093/bioinformatics/btu048 (2014). 24463181 PMC4016707

[b36] Doench J. G. *et al.* Rational design of highly active sgRNAs for CRISPR-Cas9-mediated gene inactivation. Nat Biotechnol 32, 1262–1267, doi: 10.1038/nbt.3026 (2014). 25184501 PMC4262738

[b37] Wong N., Liu W. & Wang X. WU-CRISPR: characteristics of functional guide RNAs for the CRISPR/Cas9 system. Genome Biol 16, 218, doi: 10.1186/s13059-015-0784-0 (2015). 26521937 PMC4629399

[b38] Chari R., Mali P., Moosburner M. & Church G. M. Unraveling CRISPR-Cas9 genome engineering parameters via a library-on-library approach. Nat Methods 12, 823–826, doi: 10.1038/nmeth.3473 (2015). 26167643 PMC5292764

[b39] Moreno-Mateos M. A. *et al.* CRISPRscan: designing highly efficient sgRNAs for CRISPR-Cas9 targeting *in vivo*. Nat Methods 12, 982–988, doi: 10.1038/nmeth.3543 (2015). 26322839 PMC4589495

[b40] Tebas P. *et al.* Gene editing of CCR5 in autologous CD4 T cells of persons infected with HIV. N Engl J Med 370, 901–910, doi: 10.1056/NEJMoa1300662 (2014). 24597865 PMC4084652

[b41] Carroll D. Genome engineering with zinc-finger nucleases. Genetics 188, 773–782, doi: 10.1534/genetics.111.131433 (2011). 21828278 PMC3176093

[b42] Boch J. *et al.* Breaking the code of DNA binding specificity of TAL-type III effectors. Science 326, 1509–1512, doi: 10.1126/science.1178811 (2009). 19933107

[b43] Travis J. Making the cut. Science 350, 1456–1457, doi: 10.1126/science.350.6267.1456 (2015). 26680172

[b44] Sun G. Q., Wang S. L., Ren Q., Jin Z. & Wu Y. P. Effects of time delay and space on herbivore dynamics: linking inducible defenses of plants to herbivore outbreak. Sci Rep 5, 11246, doi: 10.1038/srep11246 (2015). 26084812 PMC4471659

[b45] Sun G.-Q., Wu Z.-Y., Wang Z. & Jin Z. Influence of isolation degree of spatial patterns on persistence of populations. Nonlinear Dynamics 83, 811–819 (2016).

[b46] Sun G.-Q. *et al.* Influence of time delay and nonlinear diffusion on herbivore outbreak. Communications in Nonlinear Science and Numerical Simulation 19, 1507–1518 (2014).

[b47] Sciabola S., Cao Q., Orozco M., Faustino I. & Stanton R. V. Improved nucleic acid descriptors for siRNA efficacy prediction. Nucleic Acids Res. 41, 1383–1394, doi: 10.1093/nar/gks1191 (2013). 23241392 PMC3561943

[b48] Lu X. J., Bussemaker H. J. & Olson W. K. DSSR: an integrated software tool for dissecting the spatial structure of RNA. Nucleic Acids Res. 43, e142, doi: 10.1093/nar/gkv716 (2015). 26184874 PMC4666379

[b49] Qureshi A., Tandon H. & Kumar M. AVP-IC Pred: Multiple machine learning techniques based prediction of peptide antiviral activity in terms of half maximal inhibitory concentration (IC). Biopolymers, doi: 10.1002/bip.22703 (2015). PMC7161829 26213387

[b50] Qureshi A., Thakur N. & Kumar M. VIRsiRNApred: a web server for predicting inhibition efficacy of siRNAs targeting human viruses. J Transl Med 11, 305, doi: 10.1186/1479-5876-11-305 (2013). 24330765 PMC3878835

[b51] Lorenz R. *et al.* ViennaRNA Package 2.0. Algorithms Mol Biol 6, 26, doi: 10.1186/1748-7188-6-26 (2011). 22115189 PMC3319429

[b52] Rajput A., Gupta A. K. & Kumar M. Prediction and analysis of quorum sensing peptides based on sequence features. PLoS One 10, e0120066, doi: 10.1371/journal.pone.0120066 (2015). 25781990 PMC4363368

[b53] Thakur N., Qureshi A. & Kumar M. AVPpred: collection and prediction of highly effective antiviral peptides. Nucleic Acids Res 40, W199–W204, doi: 10.1093/nar/gks450 (2012). 22638580 PMC3394244

[b54] Sing T., Sander O., Beerenwinkel N. & Lengauer T. ROCR: visualizing classifier performance in R. Bioinformatics 21, 3940–3941, doi: 10.1093/bioinformatics/bti623 (2005). 16096348

[b55] Vacic V., Iakoucheva L. M. & Radivojac P. Two Sample Logo: a graphical representation of the differences between two sets of sequence alignments. Bioinformatics 22, 1536–1537, doi: 10.1093/bioinformatics/btl151 (2006). 16632492

