# Supplementary material for: ge-CRISPR - An integrated pipeline for the prediction and analysis of sgRNAs genome editing efficiency for CRISPR/Cas system
Source: Sci Rep. 2016 Sep 1;6:30870. doi: 10.1038/srep30870 (PMC5007494; doi:10.1038/srep30870)
Supplement: Supplementary Information [file srep30870-s1.pdf]

ge-CRISPR - An integrated pipeline for the prediction and analysis of sgRNAs genome editing efficiency for CRISPR/Cas system

Karambir Kaur[#], Amit Kumar Gupta[#], Akanksha Rajput[#] and Manoj Kumar*

Bioinformatics Centre, Institute of Microbial Technology, Council of Scientific and Industrial Research, Sector 39A, Chandigarh-160036, India

# Equal contribution

* To whom correspondence should be addressed: manojk@imtech.res.in

Karambir Kaur

Bioinformatics Centre, Institute of Microbial Technology, Council of Scientific and Industrial Research, Sector 39A, Chandigarh-160036, India, karman@imtech.res.in

Amit Kumar Gupta

Bioinformatics Centre, Institute of Microbial Technology, Council of Scientific and Industrial Research, Sector 39A, Chandigarh-160036, India, amitg@imtech.res.in

Akanksha Rajput

Bioinformatics Centre, Institute of Microbial Technology, Council of Scientific and Industrial Research, Sector 39A, Chandigarh-160036, India, akanksha@imtech.res.in

Manoj Kumar

Bioinformatics Centre, Institute of Microbial Technology, Council of Scientific and Industrial Research, Sector 39A, Chandigarh-160036, India, manojk@imtech.res.in

Supplementary information

Figure S1: Workflow of pipeline-1. (a) sgRNA scanner for extracting sgRNAs in user provided genome/gene (b) output of pilpeline-1 depicting high and low potency sgRNAs (c) sgRNA profile, displaying secondary structure and off-targets associated with individual sgRNA.

Table S1. Performance of geCRISPRc predictive models on sgRNAdesigner dataset (1841)

Table S2. Performance of geCRISPRc predictive models on sgRNAscorer (Cas_{sp}) dataset (297)

Table S3. Performance of geCRISPRc predictive models on sgRNAscorer (Cas_{st1}) dataset (171)

Table S4. Performance of geCRISPRr predictive models on CRISPRscan dataset (1020)

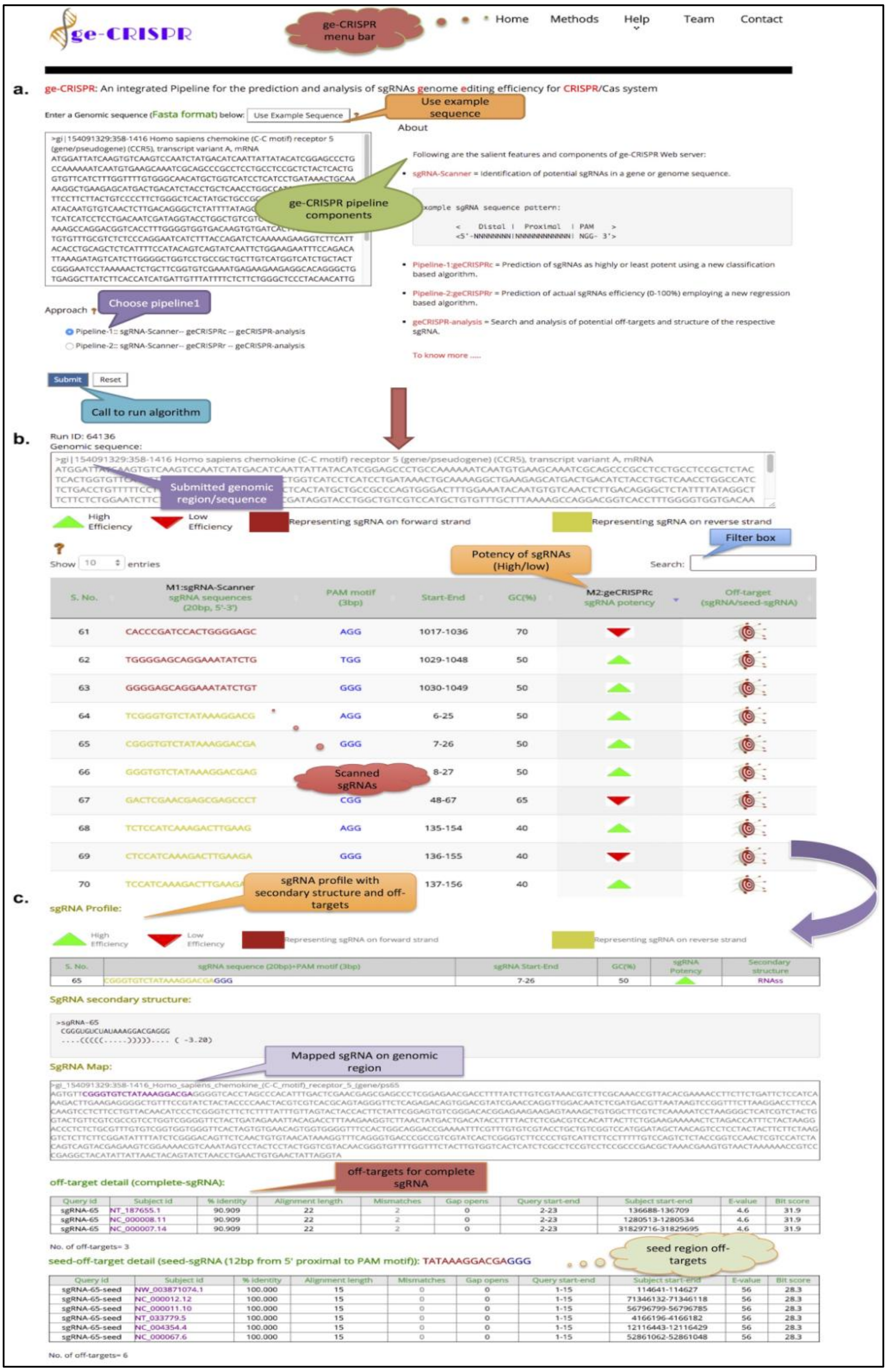

Figure S1: Workflow of pipeline-1. (a) sgRNA scanner for extracting sgRNAs in user provided genome/gene (b) output of pilpeline-1 depicting high and low potency sgRNAs (c) sgRNA profile, displaying secondary structure and off-targets associated with individual sgRNA.

Table S1: Performance of geCRISPRc predictive models on sgRNAdesigner dataset (1841)

S.No	Properties	Length	Vector	Acc	Mcc	ROC
1	Composition (mono)	20	4	68.34	0.37	0.73
2	Composition (di)	20	16	75.68	0.52	0.84
3	Composition (tri)	20	64	78.8	0.59	0.87
4	Composition (tetra)	20	256	74.32	0.49	0.82
5	Composition (penta)	20	1024	70.65	0.44	0.78
6	Binary (mono)	20	80	97.42	0.95	0.99
7	Binary (di)	20	304	97.28	0.95	1.00
8	Binary (tri)	20	1152	93.34	0.87	0.98
9	Thermodynamic	20	21	78.4	0.57	0.84
10	Secondary structure	20	20	60.19	0.24	0.63
11	1+2	20	20	87.09	0.74	0.93
12	1+2+3	20	84	85.6	0.72	0.93
13	1+2+3+4	20	340	83.97	0.69	0.93
14	1+2+3+4+5	20	1364	83.83	0.68	0.91
15	6+7	20	384	97.83	0.96	1.00
16	6+7+8	20	1536	96.88	0.94	1.00
17	1+2+3+4+5+6+7+8	20	2900	88.72	0.77	0.95
18	1+2+6+7	20	404	96.88	0.94	0.99
19	1+2+3+4+6+7	20	724	91.98	0.84	0.97
20	1+2+6+7+9	20	425	96.33	0.93	0.99
21	1+2+6+7+10	20	424	96.47	0.93	0.99
22	1+2+6+7+9+10	20	445	94.43	0.89	0.99

Acc, accuracy; MCC, Matthew’s correlation coefficient; AUC, area under curve

Table S2: Performance of geCRISPRc predictive models on sgRNAscorer (Cas_{sp}) dataset (297)

S.No	Properties	Length	Vector	Acc	Mcc	ROC
1	Composition (mono)	23	4	68.46	0.37	0.72
2	Composition (di)	23	16	70.97	0.42	0.74
3	Composition (tri)	23	64	67.03	0.34	0.70
4	Composition (tetra)	23	256	65.95	0.34	0.68
5	Composition (penta)	23	1024	62.01	0.24	0.63
6	Binary (mono)	23	92	74.91	0.53	0.83
7	Binary (di)	23	352	74.91	0.50	0.81
8	Binary (tri)	23	1344	70.61	0.46	0.80
9	Thermodynamic	23	21	64.52	0.31	0.66
10	Secondary structure	23	23	63.08	0.28	0.64
11	1+2+3+4	23	340	69.89	0.40	0.73
11	1+2+3+4+5	23	1364	70.25	0.40	0.83
13	6+7	23	444	76.70	0.53	0.73
14	6+7+8	23	1788	76.70	0.54	0.82
15	1+2+3+4+5+6+7+8	23	3152	69.89	0.40	0.73
16	1+2+3+4+6+7	23	784	70.61	0.41	0.73

Acc, accuracy; MCC, Matthew’s correlation coefficient; AUC, area under curve

Table S3: Performance of geCRISPRc predictive models on sgRNAscorer (Cas_{st1}) dataset (151)

S.No	Properties	Length	Vector	Acc	Mcc	ROC
1	Composition (mono)	27	4	74.17	0.49	0.78
2	Composition (di)	27	16	75.5	0.51	0.75
3	Composition (tri)	27	64	72.85	0.46	0.77
4	Composition (tetra)	27	256	70.2	0.45	0.73
5	Composition (penta)	27	1024	66.23	0.34	0.70
6	Binary (mono)	27	108	83.44	0.67	0.89
7	Binary (di)	27	416	79.47	0.59	0.86
8	Binary (tri)	27	1600	73.51	0.46	0.75
9	Thermodynamic	27	21	74.17	0.48	0.77
10	Secondary structure	27	27	66.89	0.39	0.65
11	1+2+3+4	27	340	73.51	0.49	0.79
12	1+2+3+4+5	27	1364	73.51	0.48	0.77
13	6+7	27	524	82.78	0.65	0.89
14	6+7+8	27	2124	82.67	0.66	0.88
15	1+2+3+4+5+6+7+8	27	3488	72.85	0.47	0.77
16	1+2+3+4+6+7	27	864	73.51	0.47	0.76

Acc, accuracy; MCC, Matthew's correlation coefficient; AUC, area under curve

Table S4: Performance of geCRISPRr predictive models on CRISPRscan dataset (1020)

S.No	Properties	Length	Vector	PCC
1	Composition (mono-di-tri-tetra)	20	340	0.42
2	Binary (mono-di)	20	384	0.43
3	1+2	20	724	0.43

PCC; Pearson correlation coefficient
